# Does access to no-cost contraception change method selection among individuals who report difficulty paying for health-related care?

**DOI:** 10.1186/s12905-022-01911-x

**Published:** 2022-08-02

**Authors:** Alexandra Gero, Rebecca G. Simmons, Jessica N. Sanders, David K. Turok

**Affiliations:** Department of Obstetrics and Gynecology, University of Utah School of Medicine, 30N 1900E Rm 2B-200, Salt Lake City, UT 84132 USA

**Keywords:** Contraception, Low-cost, Healthcare, Poverty

## Abstract

**Background:**

Out-of-pocket costs continue to be a barrier to accessing necessary healthcare services, including contraception. We explored how eliminating out-of-pocket cost affects contraceptive method choice among people reporting difficulty paying for healthcare in the previous year, and whether method satisfaction differed by method choice.

**Methods:**

We used data from the HER Salt Lake Contraceptive Initiative. This prospective cohort study provided participants with no-cost contraception (April 2016–March 2017) following a control period that provided no reduction in cost for the contraceptive implant, a reduced price for the hormonal IUD, and a sliding scale that decreased to no-cost for the copper IUD (September 2015–March 2016). We restricted the study population to those who reported difficulty paying for healthcare in the past 12 months. For our primary outcome assessing changes in method selection between intervention and control periods, we ran simultaneous multivariable logistic regression models for each method, applying test corrections for multiple comparisons. Among participants who continued their method for 1 year, we explored differences in method satisfaction using multivariable logistic regression.

**Results:**

Of the 1,029 participants reporting difficulty paying for healthcare and controlling for other factors, participants more frequently selected the implant (aOR 6.0, 95% CI 2.7, 13.2) and the hormonal IUD (aOR 3.2, 95% CI 1.7, 5.9) during the intervention than control period. Comparing the same periods, participants less frequently chose the injection (aOR 0.5, 95% CI 0.3, 0.8) and the pill (aOR 0.4, 95% CI 0.3, 0.6). We did not observe a difference in uptake of the copper IUD (aOR 2.0, 95% CI 1.0, 4.1).Contraceptive satisfaction scores differed minimally by contraceptive method used among contraceptive continuers (n = 534). Those who selected LNG IUDs were less likely to report low satisfaction with their method (aOR 0.5, 95% CI 0.3, 0.97).

**Conclusion:**

With costs removed, participants who reported difficulty paying for healthcare were more likely to select hormonal IUDs and implants and less likely to select the injectable or contraceptive pills. Among continuers, there were few differences in method satisfaction.

*ClinicalTrials.gov Identifier* NCT02734199

**Supplementary Information:**

The online version contains supplementary material available at 10.1186/s12905-022-01911-x.

## Background

Uptake of long-acting reversible contraceptive (LARC) methods in the United States has increased dramatically over the last two decades, especially in the years since enacting the Affordable Care Act’s (ACA) contraceptive coverage mandate [1]. While this coverage has made LARC methods more affordable for many [2], there is still evidence that in states with imperfect implementation of the ACA, many others still have difficulty affording the highly effective methods that they want. A post-ACA evaluation found that more than 200,000 women^1^ in Utah were still in need of subsidized family planning services [3]. Results from contraceptive access initiatives across the nation, indicate that removing costs leads to increased utilization of LARCs [4–7]. Utah’s HER Salt Lake Contraceptive Initiative (HER Salt Lake) found the odds of LARC uptake to increase as much as 2.5 times durng the intervention period compared to the control period [5].

HER Salt Lake launched in 2015 through a partnership between the University of Utah Family Planning Division and Planned Parenthood Association of Utah (PPAU), and assessed the impact of removing cost on method selection for eligible study subjects in Salt Lake County, Utah [5]. In this secondary analysis, we explored the impact of cost removal on the subset of HER Salt Lake study participants reporting trouble paying for health-related care. Additionally, recognizing that cost is only one factor that may influence method satisfaction (or dissatisfaction), we assessed whether satisfaction differed by method selected across study periods (routine care vs. provision of no-cost contraceptive care for all methods.

## Methods

### Study population

HER Salt Lake is a prospective quasi-experimental observational cohort study that enrolled eligible women at four participating Planned Parenthood Association of Utah (PPAU) clinics in Salt Lake County, between September 2015 and March 2017 [5]. Prior to beginning the control period, staff at all PPAU health centers throughout the state were trained to offer clients standardized, patient-centered counseling; the method effectiveness chart and counseling discussion guide used during these counseling sessions are available as Additional file 1 and 2. During the six-month control period and consistent with clinic protocol prior to study implementation, contraceptive clients with incomes below 250% federal poverty level (FPL) received low-cost, but not free, care. During this time the copper IUD was available on a sliding cost scale that went down to no-cost and the levonorgestrel 52 mg IUS, Liletta® (distributed by Allergan/Medicines360) was available through 340b, the federally-sponsored medication assistance program, for a total device cost of $50. The contraceptive implant was not available at reduced cost. Following the control period, a 12-month intervention period removed all out-of-pocket contraceptive services costs for three years starting with a patient’s enrollment visit, regardless of the patient’s income. Those receiving care during the no-cost intervention period could return as often as they liked for three years and switch to any other method without cost. Details of HER Salt Lake study eligibility and methodology are reported elsewhere [5].

### Data collection

**Fig. 1:**
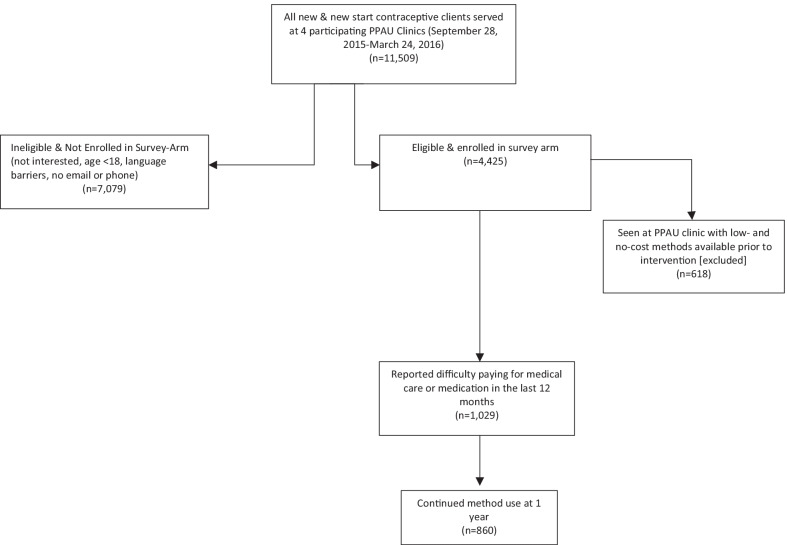
Participant flow chart for study sample of women reporting difficulty paying for health care among HER Salt Lake participants

To be eligible for the prospective survey arm of the study, patients had to (1) be between 18 and 45 years; (2) be fluent in English or Spanish; (3) desire to prevent pregnancy for at least one year; (4) have a working mobile phone; and (5) have incomes under 300% FPL. During the 18-month study period, 4,425 patients consented to participate in the prospective study and agreed to complete detailed questionnaires at enrollment and eight subsequent timepoints (1-, 3-, 6-, 12-, 18-, 24-, 30- and 36-months post-enrollment). This secondary analysis is restricted to HER Salt Lake prospective study participants who answered ‘Yes’ to the question “In the past 12 months, have you had trouble paying for medical care or medications?” in the enrollment survey. The one participating abortion clinic offered low- and no-cost contraception prior to the inititation of HER Salt Lake; we thus excluded participants served at this clinic from this analytic sample. Selection of our study sample is detailed in Fig. 1. We utilized both survey data and medical record data. We collected and stored survey data through the secure, web-based Research Electronic Data Capture (REDCap). We extracted participants’ health records, including contraceptive method selected at enrollment and changes in contraceptive method during the course of the study, from the PPAU electronic medical record system and linked these data to enrollment data.

### Statistical methods and analyses

Our primary outcome assessed changes in method selection between the control and intervention periods among HER participants that reported difficulty paying for healthcare, asked as a Yes/No question. Our secondary outcome compared one-year method satisfaction among contraceptive continuers. We compared baseline differences in participant demographics between the control and intervention period using chi-square tests and two-sample t-tests. Our baseline comparison presents the complete list of race and ethnicity categories found on the HER Salt Lake study enrollment form; due to small numbers, we collapsed these categories in our primary and secondary analyses using methodology consistent with previously published literature from the HER Salt Lake study [5]. Although we included all methods in our denominator, both primary and secondary analyses assess only the six most popular contraceptive methods (copper and hormonal IUDs, contraceptive implant, contraceptive shot, vaginal ring, and oral contraceptive pills) due to low rate of selection of other methods (2.6%).

We assessed our primary outcome by conducting simultaneous multivariate logistic regression models comparing differences in method uptake by study period and across methods. To develop our full model, we first conducted unadjusted regression analyses on all variables proposed for inclusion in the final multivariable models. We used a cut-off of 0.25 to determine covariate inclusion in the final models, as is supported by literature [8, 9]. Our covariates included variables known to influence contraceptive choice, including age, race and ethnicity, education, employment status, insurance type, federal poverty level, and parity. Additionally, we controlled for health center enrollment site, ever-use of LARC, and history of abortion, as these were significant in unadjusted analyses. Upon determining our final covariates, we ran six multivariable logistic models to assess predictors of method selection for each of the six most popular contraceptive methods. Accordingly, we applied the Benjamini–Hochberg Procedure as a test correction for multiple comparisons.

For our secondary outcome looking at method satisfaction among contraceptive continuers at one year, we defined “continuers” as those who reported continuation of same method selected at enrollment in their three-, six-, and twelve-month follow-up surveys. We made an exception when participants reported using male or female condoms, fertility awareness-based methods, withdrawal, or emergency contraception in these follow-up surveys: if a participant reported using any of these methods but later reported using the same method they selected at enrollment, we categorized them as a continuer with supplemental method use.

To assess predictors of method satisfaction within this cohort, we utilized reported method satisfaction at the 12-month survey (measured on a Likert scale with the choices completely satisfied, somewhat satisfied, neutral, somewhat dissatisfied, and completely dissatisfied). To ensure sufficient numbers for analyses, we aggregated responses into three categories: completely satisfied, somewhat satisfied/neutral, somewhat/completely dissatisfied, and compared distribution of responses in the original categories and our aggregated categories. We conducted a single multivariable model assessing predictors of method satisfaction among continuers. We hypothesized that users using the same method for one year would be ‘completely satisfied’ with that method, and therefore used this category as our referent. We performed all analyses in Stata 15.0 or higher (StataCorp LP, College Station, TX). The Unviersity of Utah IRB approved this study.

## Results

**Fig. 2:**
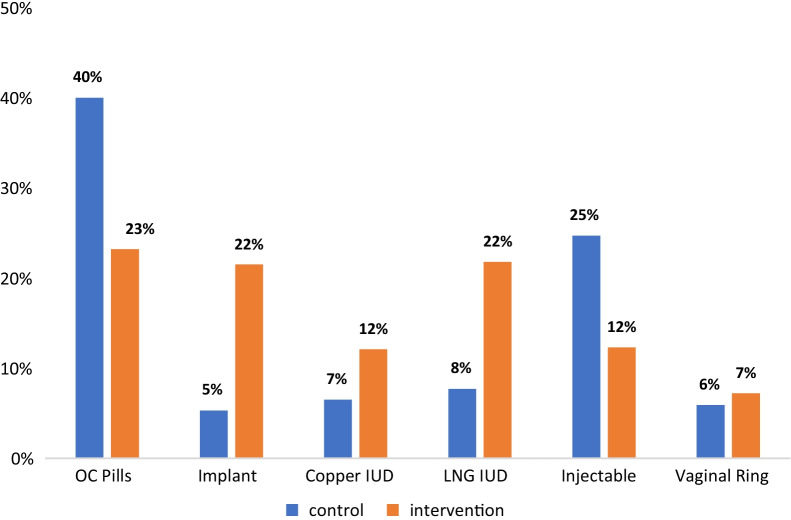
Changes in contraceptive method selection across study periods

A total of 1,029 individuals seen at the three clinics included in our sample reported difficulty paying for healthcare over the past year. This accounts for 26.9% (n = 170) of all those enrolled in the control period and 27.1% (n = 859) of all intervention subjects. Participant demographics did not differ significantly between study periods (Table 1). Nearly one-third of our sample reported incomes above the federal poverty level, indicating that this sample represents a wide range of socioeconomics statuses. Chi-square tests assessing the difference in method selection between study periods identified significant differences for each method except the vaginal ring. Distribution of method selection across study periods is highlighted in Fig. 2.

**Table 1:** Participant characteristics

Variable	Control	Intervention	*p*-value
No. (%)	(n = 170)	(n = 859)	
Age, years			0.338
18–19	22 (12.9)	129 (15.0)	
20–24	81 (47.7)	352 (41.0)	
25–29	42 (24.7)	216 (25.2)	
30–34	17 (10.0)	89 (10.4)	
35 +	8 (4.7)	73 (8.5)	
Race/Ethnicity			0.874
American Indian or Alaska Native	2 (1.2)	12 (1.4)	
Asian	3 (1.8)	22 (2.6)	
Black	4 (2.4)	29 (3.4)	
Hispanic or Latine	33 (19.5)	163 (19.2)	
Native Hawaiian or Pacific Islander	0	7 (0.8)	
White, non-Latine	112 (66.3)	542 (63.7)	
Other	15 (2.4)	76 (8.9)	
Education			0.984
High school or less	88 (52.4)	447 (52.5)	
Any college	80 (47.6)	405 (47.5)	
Employment status			0.739
Full or part-time	102 (60.7)	506 (60.2)	
Student	22 (13.1)	113 (13.4)	
Out of workforce	8 (4.8)	58 (6.9)	
Unemployed	36 (21.4)	164 (19.5)	
Federal poverty level			0.426
< 138%	124 (73.8)	598 (70.8)	
≥ 138%	44 (26.2)	247 (29.2)	
Insurance type			0.514
Private	44 (28.2)	204 (24.7)	
Public	7 (4.5)	29 (3.5)	
None	105 (67.3)	592 (71.8)	
Parity			0.492
Nulliparous	118 (69.4)	573 (66.7)	
Parous	52 (30.6)	286 (33.3)	
Ever-use of LARC			0.207
Yes	44 (25.9)	264 (30.7)	
No	126 (74.1)	595 (69.3)	
History of abortion			0.155
Yes	30 (17.8)	115 (13.6)	
No	139 (82.3)	733 (86.4)

### Predictors of method selection

We found differences in method selection at enrollment between study periods in our unadjusted logistic regression models: participants in the intervention period had increased odds of selecting the implant, the copper IUD, and the hormonal IUD compared to the control period. Conversely, participants selected oral contraceptive pills or the injectable less frequently in the intervention period than the control period. Table 2 presents unadjusted results for all methods.

**Table 2:** Unadjusted logistic regression results comparing contraceptive method selection at enrollment between intervention to control period

Method	Odds Ratio (OR) [95% CI]
Implant	4.4** [2.4, 8.3]
Copper IUD	2.2* [1.2, 4.1]
Hormonal IUD	3.3** [2.0, 5.5]
Contraceptive Pills	0.4** [0.3, 0.6]
Injectable	0.4** [0.3, 0.6]
Vaginal Ring	1.0 [0.5, 1.9]

Exponentiated coefficients; 95% confidence intervals in brackets

*p < 0.01, **p < 0.001

In adjusted models, the relationship between study period and certain LARC devices strengthened: compared to the control period, we observed a six-fold increase in the likelihood of selecting an implant during the intervention period (adjusted odds ratio (aOR) 6.0, 95% Confidence Interval (CI): 2.7, 13.2), and a three-fold increase in the likelihood of selecting a hormonal IUD during the intervention period (aOR 3.2, 95% CI: 1.7, 5.9). The relationship between selection of the copper IUD and study period lost significance in our adjusted model.

Consistent with unadjusted models, we found decreased odds of choosing oral contraceptive pills and the injectable in the intervention period compared to the control period in adjusted models (aOR 0.4, 95% CI: 0.3, 0.6), and similarly observed decreased odds of selecting injectable contraception (aOR 0.5, 95% CI: 0.3, 0.8). Once again, the likelihood of selecting the vaginal ring did not change significantly between study periods.

Results from all adjusted models are presented in Table 3. Compared to Latine participants, non-Latine, white participants had lower odds of choosing an implant (aOR 0.5, 95% CI: 0.3, 0.8), and had higher odds of choosing a hormonal IUD (aOR 1.9, 95% CI: 1.2, 3.0). Participants who reported any previous use of a LARC method were more likely to select a hormonal IUD (aOR 1.7, 95% CI: 1.2, 2.5), but not the copper IUD nor the implant. Participants without insurance were more likely to choose the pill during the intervention period compared to those with private insurance (aOR 2.2, 95% CI: 1.5, 3.3).

**Table 3:** Factors associated with method selection in simultaneous multiple regression models

	Implant	Copper IUD	Hormonal IUD	Contraceptive Pills	Injectable	Vaginal Ring
	aOR [95% CI]	aOR [95% CI]	aOR [95% CI]	aOR [95% CI]	aOR [95% CI]	aOR [95% CI]
Study period						
Control	(Referent)					
Intervention	6.00 [2.72, 13.24]^***^	2.02 [0.98, 4.14]	3.15 [1.68, 5.90]^***^	0.43 [0.29, 0.63]^***^	0.47 [0.30, 0.75]^**^	1.40 [0.62, 3.19]
Enrollment Site^a^						
Clinic 1	(Referent)					
Clinic 2	1.32 [0.88, 1.99]	0.68 [0.42, 1.10]	0.74 [0.50, 1.11]	0.94 [0.66, 1.34]	1.71 [1.06, 2.76]^*^	0.98 [0.49, 1.96]
Clinic 3	0.92 [0.57, 1.49]	0.62 [0.35, 1.11]	1.02 [0.66, 1.58]	0.88 [0.59, 1.32]	1.29 [0.74, 2.25]	2.51 [1.29, 4.90]^**^
Race & Ethnicity						
Latine (with any other race)	(Referent)					
Non-White, Other, non-Latine	0.56 [0.31, 1.01]	1.39 [0.69, 2.81]	1.15 [0.59, 2.23]	0.84 [0.49, 1.46]	1.70 [0.85, 3.40]	1.42 [0.62, 3.24]
White, Non-Latine	0.50 [0.33, 0.76]^***^	0.93 [0.53, 1.61]	1.86 [1.16, 2.98]^**^	1.09 [0.74, 1.60]	1.51 [0.90, 2.56]	0.65 [0.33, 1.26]
Education level						
High school or less	(Referent)					
Any college	0.91 [0.61, 1.34]	0.98 [0.61, 1.56]	1.26 [0.86, 1.83]	1.26 [0.90, 1.75]	0.70 [0.46, 1.08]	0.85 [0.47, 1.54]
Employment status						
Full- or part-time	(Referent)					
Student	0.98 [0.57, 1.68]	0.81 [0.42, 1.58]	1.27 [0.77, 2.09]	0.93 [0.58, 1.47]	0.91 [0.48, 1.76]	1.32 [0.60, 2.86]
Out of Workforce^b^	0.90 [0.40, 2.00]	1.46 [0.67, 3.19]	0.49 [0.20, 1.16]	0.78 [0.39, 1.58]	1.61 [0.77, 3.39]	1.21 [0.39, 3.74]
Unemployed	1.17 [0.74, 1.85]	0.55 [0.29, 1.05]	0.85 [0.53, 1.37]	1.13 [0.76, 1.69]	1.26 [0.77, 2.08]	1.00 [0.48, 2.10]
Insurance type						
Private	(Referent)					
Medicaid or Medicare	1.34 [0.53, 3.40]	0.86 [0.26, 2.90]	0.59 [0.19, 1.87]	1.55 [0.59, 4.05]	1.09 [0.41, 2.92]	1.00 [1.00, 1.00]
None	0.71 [0.47, 1.08]	0.75 [0.45, 1.24]	0.90 [0.61, 1.35]	2.19 [1.48, 3.25]^***^	0.70 [0.44, 1.14]	0.93 [0.50, 1.75]
Federal poverty level (FPL)						
Up to 138%	(Referent)					
138% and greater	1.15 [0.76, 1.72]	0.58 [0.34, 0.99]^*^	1.37 [0.94, 2.00]	1.11 [0.78, 1.57]	0.53 [0.32, 0.87]^*^	1.53 [0.85, 2.75]
Parity						
Nulliparous	(Referent)					
Parous	1.18 [0.74, 1.88]	0.97 [0.55, 1.73]	1.49 [0.95, 2.34]	0.74 [0.49, 1.13]	0.99 [0.59, 1.65]	0.93 [0.46, 1.88]
Ever-use of LARC						
No	(Referent)					
Yes	1.02 [0.68, 1.53]	1.50 [0.93, 2.41]	1.70 [1.17, 2.48]^**^	0.62^*^ [0.43, 0.89]	0.49 [0.30, 0.80]^**^	0.92 [0.49, 1.71]
Age category						
18–19	(Referent)					
20–24	1.15 [0.67, 1.95]	1.06 [0.52, 2.16]	0.96 [0.55, 1.68]	1.17 [0.73, 1.87]	1.00 [0.54, 1.88]	1.05 [0.42, 2.62]
25–29	0.97 [0.53, 1.77]	1.35 [0.62, 2.95]	0.75 [0.41, 1.40]	0.96 [0.57, 1.64]	1.49 [0.75, 2.99]	1.68 [0.64, 4.42]
30–34	0.50 [0.21, 1.18]	1.60 [0.63, 4.03]	0.91 [0.43, 1.93]	0.88 [0.45, 1.74]	1.88 [0.83, 4.26]	1.62 [0.49, 5.31]
35 +	0.41 [0.16, 1.05]	0.96 [0.32, 2.85]	1.43 [0.64, 3.17]	1.21 [0.57, 2.60]	1.53 [0.60, 3.92]	1.61 [0.41, 6.38]
History of abortion						
No	(Referent)					
Yes	0.69 [0.39, 1.23]	1.22 [0.68, 2.18]	1.12 [0.69, 1.83]	0.80 [0.50, 1.27]	1.30 [0.77, 2.21]	1.36 [0.65, 2.84]

Exponentiated coefficients; 95% confidence intervals in brackets

*p < 0.05, **p < 0.01, ***p < 0.001

^a^Clinic 1 represents PPAU’s Salt Lake location, 2 is the West Valley City clinic, 3 is the South Jordan clinic

^b^Includes participants who reported they are on leave, retired, homemakers, or “other”

### Method satisfaction

Of the 860 participants who provided one year of method use data, 62.1% (n = 534) reported using the same method continually since enrollment. We found the highest continuation rates among hormonal and copper IUD users, and contraceptive implant users (78.1% 76.9% and 68.1%, respectively). We observed lower continuation rates for the injectable, the vaginal ring, and the pill (55.5%, 44.3%, and 49.1%, respectively).

Compared to contraceptive pill continuers, continuers of the hormonal IUD were less likely feel neutral or somewhat satisfied with their method. Participants in their late 20 s were less likely to feel dissatisfied with their method than those in other age groups, while participants with a history of abortion were more likely to feel dissatisfactied. Participants in both their late 20 s and late 30 s were also less likely to feel somewhat satisfied with their method than those in other age groups. Table 4 presents unadjusted model results, and Table 5 details the results of our adjusted model.

**Table 4:** Unadjusted logistic regression results comparing method satisfaction across methods among continuers

	Not Satisfied (n = 40)	Somewhat Satisfied or Neutral (n = 148)
Method Selected	OR [95% CI]	OR [95% CI]
Contraceptive Pills	(Referent)	
Implant	0.6 [0.2, 17]	0.9 [0.5, 1.6]
Copper IUD	0.9 [0.3, 2.4]	0.7 [0.5, 1.3]
Hormonal IUD	0.4 [0.2, 1.2]	0.6 [0.3, 1.0]
Injectable	0.2 [0.05, 1.1]	0.5 [0.2, 1.0]
Vaginal Ring	0.3 [0.2, 1.2]	0.5 [0.2, 1.3]
Study period		
Control	(Referent)	
Intervention	3.0 [0.7, 13.4]	2.3 [1.1, 4.7]

*p < 0.05, **p < 0.01, ***p < 0.001

**Table 5:** Predictors of being less than completely satisfied at one-year among continuers

	Not satisfied at one year (n = 40)		Somewhat satisfied or neutral at one year (n = 148)	
	aOR	95% Confidence Interval	aOR	95% Confidence Interval
Method				
Hormonal implant	0.49	[0.14, 1.66]	0.85	[0.44, 1.64]
Copper IUD	0.99	[0.32, 3.09]	0.64	[0.30, 1.35]
Depo shot	0.19	[0.04, 1.06]	0.45	[0.20, 1.01]
Vaginal ring	0.24	[0.02, 2.45]	0.39	[0.13, 1.19]
LNG IUDs	0.38	[0.12, 1.19]	0.51^*^	[0.27, 0.97]
OC Pills	(Referent)			
Study period				
Control	(Referent)			
Intervention	2.51	[0.52, 12.03]	1.97	[0.91, 4.26]
Enrollment Site^a^				
Clinic 1	(Referent)			
Clinic 2	0.66	[0.27, 1.62]	0.72	[0.43, 1.20]
Clinic 3	1.01	[0.39, 2.64]	0.91	[0.53, 1.57]
Race/Ethnicity				
Latine (with any other race)	(Referent)			
Non-White, Other, non-Latine	0.89	[0.20, 4.03]	0.96	[0.42, 2.18]
White, non-latine	0.90	[0.31, 2.61]	0.89	[0.50, 1.59]
Insurance type				
Private	(Referent)			
Medicaid or medicare	1.68	[0.21, 13.31]	0.94	[0.20, 4.41]
None	0.93	[0.39, 2.24]	1.08	[0.66, 1.76]
Federal poverty level				
Up to 138%	(Referent)			
138% and greater	1.11	[0.47, 2.62]	1.36	[0.84, 2.20]
Parity				
Nulliparous	(Referent)			
Parous	0.96	[0.34, 2.66]	1.55	[0.88, 2.75]
Ever-Use of LARC				
No	(Referent)			
Yes	1.28	[0.54, 3.05]	0.97	[0.58, 1.61]
Age category				
18–19	(Referent)			
20–24	0.60	[0.19, 1.85]	0.62	[0.30, 1.28]
25–29	0.18^*^	[0.04, 0.80]	0.44^*^	[0.19, 0.98]
30–34	1.22	[0.28, 5.29]	1.00	[0.38, 2.59]
35 +	0.26	[0.03, 1.96]	0.27^*^	[0.08, 0.93]
History of abortion				
No	(Referent)			
Yes	3.45^*^	[1.28, 9.34]	1.46	[0.73, 2.92]
Education level				
High school or less	(Referent)			
Any college	0.81	[0.36, 1.83]	1.61	[0.99, 2.60]
Employment status				
Full- or part-time	(Referent)			
Student	0.00	[0.00, .]	1.11	[0.61, 2.03]
Out of workforce^b^	0.60	[0.10, 3.55]	1.24	[0.49, 3.19]
Unemployed	1.53	[0.55, 4.25]	1.06	[0.54, 2.06]

Exponentiated coefficients; 95% confidence intervals in brackets

*p < 0.05

^a^Clinic 1 represents PPAU’s Salt Lake location, 2 is the West Valley City clinic, 3 is the South Jordan clinic

^b^Includes participants who reported they are on leave, retired, homemakers, or “other”

## Discussion

Adjusted models for our primary outcome demonstrated distinct findings for changes in uptake of each LARC method when patient costs were completely removed during the intervention period. The copper IUD was the one LARC method available for some people at no cost during the control period and did not increase in use during the intervention period. The hormonal IUD, offered at a reduced cost during the control period, saw a three-fold increase in selection during the intervention. Meanwhile, the only LARC method not available at a reduced cost in the control period, the contraceptive implant, showed a six-fold increase in selection with cost removal. These changes in the primary outcome make economic sense at the individual level and align with existing literature demonstrating the significant influence method cost has on contraception method selection [4, 5].

In this study, we assessed the effect of removing cost for contraceptive care on method selection exclusively among study participants reporting prior difficulty paying for healthcare and healthcare-related services. With cost removal, people more frequently chose the implant and hormonal IUD, and less frequently chose oral contraceptive pills and the injectable.

Importantly, we found that reports of difficulty paying for healthcare were not limited by socioeconomic status. In fact, 29% of our sample reported incomes above the federal poverty line, and 38% of the entire HER Salt Lake cohort reported incomes between 101–300% FPL [10]. It is widely recognized that healthcare, and prescription drugs specifically, is prohibitively expensive for many Americans [11–13]; this may explain why we did not see hypothesized differences in method selection when comparing our subset of participants reporting to the larger enrolled population during either study period. This finding also adds to the existing body of literature indicating that reducing cost barriers helps to ameliorate healthcare disparities [14].

The 340B drug pricing program, administered by the federal government, is meant to help non-profit hospitals and clinics purchase outpatient medications at reduced costs [15]. During the control period, the hormonal IUD was available at reduced cost through the 340B drug pricing program; participants paid for the insertion, plus $50 for the device itself. In spite of this, we still observed an increased rate of hormonal IUD selection when it was available at no cost during the intervention period. This increase, however, was much smaller in magnitude than that of the contraceptive implant, which was not available at a reduced price during the control period.

Our results, similar to results of prior research, suggest that more people choose these methods when cost is not a barrier [4, 5, 16]. Participants in our study were twice as likely to choose a hormonal IUD with removal of costs during the intervention period as compared to the control period. However, the six-fold increase we found in selection of the contraceptive implant during the intervention period suggests high demand for an affordable option for this device. After a July 2020 Supreme Court ruling undermined the ACA’s contraceptive coverage mandate by allowing employers to limit access to contraception under their employer-sponsored insurance plan, the dearth of ‘low cost’ LARC methods on the market is especially relevant [17].

Similar to other contraceptive initiatives such as CHOICE, HER Salt Lake participants could select from the full range of contraceptive methods at enrollment free of charge [4, 5]. Any HER Salt Lake participant was also able to switch methods at no cost, and as frequently as desired, during the duration of the study’s intervention period. While we observed higher one-year continuation rates among those who selected a LARC device than those who chose a short-acting method, we found very few differences in method satisfaction among contraceptive continuers. Future research could explore the types of considerations that impact method continuation.

Strengths of this study include its large cohort who provided baseline information and prospectively provided regular follow-up data. Participating clinics are all in the same health system and staff at all clinics received the same contraceptive counseling training prior to the control period. Another strength is use of both EHR and survey data to assess method selection, as these data sources allow for data quality assurance.

Our study findings may be limited by the fact that participants self-identified as having difficulty paying for healthcare. The concept of “difficulty” was not specifically defined, meaning the implications of this categorization are likely broad. Further research is needed to more accurately define how different difficulties in paying for healthcare may manifest with respect to contraceptive access. Additionally, survey data is not well-suited to the task of teasing apart the factors that contribute to someone’s subjective definition of satisfaction broadly, and specifically what it means to be satisfied with their contraceptive method. Relying on these data could potentially lead to misclassification of method satisfaction results. Conducting HER Salt Lake in the urban area of Utah possibly limits the generalizability of our findings to people outside of Utah, or people living in rural areas. We do note that two-thirds of our population identified as non-Latine white, reflective of the 61% of the U.S. population in that group.

## Conclusion

Despite the existence of sliding scale fees and patient assistance programs in the control period, a no-cost contraceptive intervention still increased utilization for long-acting methods, specifically hormonal IUDs and implants, among study participants who reported prior difficulty paying for healthcare. Additionally, method satisfaction did not differ across methods among participants with continued method use. Finally, and perhaps most importantly, people across income levels report difficulty paying for healthcare.

## Supplementary Information

**Additional file 1:** Method Effectiveness Chart.

**Additional file 2:** Counseling Discussion Guide.

**Additional file 3:** De-identified dataset used in analyses.

## Data Availability

A de-identified dataset of the data generated and analyzed during this study is included in this published article and its supplementary information files (Additional file 3).

## Abbreviations

LARC: Long-acting removable contraception
ACA: Affordable Care Act
HER Salt Lake: HER Salt Lake Contraceptive Initiative
PPAU: Planned Parenthood Association of Utah
FPL: Federal poverty level
REDCap: Research Electronic Data Capture
IUD: Intrauterine Device
OR: Odds Ratio
aOR: Adjusted Odds Ratio
CI: Confidence Interval

## Footnotes

1 We use the term “women” and participants interchangeably throughout this article but acknowledge that not all individuals who need contraceptive services (or who were served by this initiative) identify as women. Transgender men and gender-nonconforming individuals were eligible for all aspects of participation.
