# Supplementary material for: Does access to no-cost contraception change method selection among individuals who report difficulty paying for health-related care?
Source: BMC Womens Health. 2022 Aug 2;22:327. doi: 10.1186/s12905-022-01911-x (PMC9344653; doi:10.1186/s12905-022-01911-x)
Supplement: Supplementary file 1 — Additional file 1: Method Effectiveness Chart. [file 12905_2022_1911_MOESM1_ESM.docx]

Appendix 1. Method Effectiveness Chart used in HER Salt Lake Contraceptive Counseling Visits

**Choosing a Method of Birth Control**

| **Group A**  (provider sets it and you forget it) | Implants 🟊 🟊 🟊 🟊  IUD (Hormonal) 🟊 🟊 🟊 🟊  IUD (Non-hormonal) 🟊 🟊 🟊 🟊 |
| --- | --- |
| **Group B**  (once every 3 months, monthly, weekly, daily) | Shot (Depo) 🟊 🟊 🟊  Vaginal Ring 🟊 🟊 🟊  Patch 🟊 🟊 🟊  Pill 🟊 🟊 🟊 |
| **Group C**  (must use every single intercourse) | Diaphragm 🟊 🟊  Sponge 🟊 🟊  Male Condom 🟊 🟊  Cervical Cap 🟊 🟊  Female Condom 🟊 🟊  Rhythm Method 🟊  Withdrawal 🟊  Spermicides 🟊 |

Approximate effectiveness: 🟊 🟊 🟊 🟊 = 99% 🟊 🟊 🟊 = 91% 🟊 🟊 = 85% 🟊 = 75%

(Sterilization is not included in the chart as it is only for women who completely rule out the possibility of ever wanted to have (more) children in the future.)

**Elección de un Método de Control de Natalidad**

| **Grupo A**  (proveedormedico lo coloca y usted se olvida) | Implantes 🟊 🟊 🟊 🟊  Dispositivo Intrauterino (Hormonal) 🟊 🟊 🟊 🟊  Dispositivo Intrauterino (Sin hormonas) 🟊 🟊 🟊 🟊 |
| --- | --- |
| **Grupo B**  (una vez cada 3 meses, mensual, semanal, diaria) | Inyección (Depo) 🟊 🟊 🟊  Anillo Vaginal 🟊 🟊 🟊  Parche 🟊 🟊 🟊  La Pildora 🟊 🟊 🟊 |
| **Grupo C**  (debe usarlo en cada relación sexual) | Diafragma 🟊 🟊  Esponja 🟊 🟊  Condón Masculino 🟊 🟊  Capuchón Cervical 🟊 🟊  Condón Femenino 🟊 🟊  Método de Ritmo 🟊  Retirada 🟊  Espermicidas 🟊 |

Efectividad Aproximada: 🟊 🟊 🟊 🟊 = 99% 🟊 🟊 🟊 = 91% 🟊 🟊 = 85% 🟊 = 75%

(Le esterilización no está includida en esta table, ya que es solo para mujeres que han descartado por completo la posibilidad de tener (más) hijos en el futuro.)
