# Supplementary material for: Does access to no-cost contraception change method selection among individuals who report difficulty paying for health-related care?
Source: BMC Womens Health. 2022 Aug 2;22:327. doi: 10.1186/s12905-022-01911-x (PMC9344653; doi:10.1186/s12905-022-01911-x)
Supplement: Supplementary file 2 — Additional file 2: Counseling Discussion Guide. [file 12905_2022_1911_MOESM2_ESM.docx]

Appendix 2. Discussion Guide used in HER Salt Lake Contraceptive Counseling Sessions

**INTRODUCTION**

**-**Hi my name is ______and I am a Clinical Assistant.

-We are experts & we are here for you. Call us anytime and I or one of my teammates will be happy to help. Here’s a card with our phone number & my name on it. {Give card with your first name written on it.}

**CHART**

**-**We want to help you make the best decision for you. This is a chart of all the birth control options available here. They are organized into three groups.

**-**Group A methods are the best at preventing pregnancy and easiest to use - our provider puts in place for you & then you don’t do anything until or if you want to be pregnant. Group B are also very good at preventing pregnancy when used correctly and you have to do something daily, weekly, or monthly to make them work. Group C are things you have to do every time you have sex and are not as good at preventing pregnancy.

**-**What questions do you have about the methods chart? {Wait 7 seconds.}

-What thoughts do you have about a possible method?

**HAVE THE CONVERSATION**

1. What do you like about this method? What do you dislike?
2. How would people important to you, like your partner, family or friends feel about you using this method?
3. How does this method fit with who you are-your lifestyle? How does this method fit with how you see yourself?

4. If you decided to use this method, how easy or hard would be to use it correctly?

5. What feelings do you have about this method?

**INFORMATION SHEETS**

**-**Let’s go over the information sheets we have for the method ______. You will get to take this with you, but let’s talk about a few sections.

**-**These are the side effects. Most side effects are temporary, usually lasting 2-3 months.

-Which of these side effects might be hard for you personally to deal with? What would you do to deal with that?

-For a person on method for awhile “I know you have been using ___ for awhile. Which of these side effects might be hard for you to deal with, if they happened now?”

**ACCURATE USE – Group B or C only**

**-**What is your plan to be sure you take your pill every day? (or put in your ring on time or come back in for your next shot or change your patch).

**-**What would your back-up plan be, like if you miss a pill/ring/shot? {Discuss emergency contraception.}

**-**How are you going to get refills? How will you remember to get them?

**SWITCH METHODS**

**-**What would you do if you wanted to switch your method? {Use the word gap. Say: Don’t stop using your method. When there are gaps in your birth control, you can become pregnant.}

**Condom Use**

-The ____ method is great at preventing pregnancy but does not prevent STDs so make sure you use condoms if your worried about that.

**Pregnancy Plans**

You stated earlier that you unsure of planning a pregnancy in the next year. Being unsure about wanting to become pregnant makes you use your method less accurately, so be extra careful.
